# Synthesis of Carbon Nanotube Arrays with High Aspect Ratio via Ni-Catalyzed Pyrolysis of Waste Polyethylene

**DOI:** 10.3390/nano8070556

**Published:** 2018-07-21

**Authors:** Yangfan Zheng, Haijun Zhang, Shengtao Ge, Jianbo Song, Junkai Wang, Shaowei Zhang

**Affiliations:** 1 The State Key Laboratory of Refractories and Metallurgy, Wuhan University of Science and Technology, Wuhan 430081, China; 15671628617@163.com (Y.Z.); 13006396682@163.com (S.G.); 13129967187@163.com (J.S.); jkwang0914@163.com (J.W.); 2 College of Engineering, Mathematics and Physical Sciences, University of Exeter, ExeterEX4 4QF, UK

**Keywords:** carbon nanotubes, nickel dichloride, waste polyethylene, catalytic pyrolysis, adsorption, magnetically recycle

## Abstract

Carbon nanotube (CNT) arrays 30–50 nm in diameter and with a length of several micrometers were prepared by catalytic pyrolysis of waste polyethylene in Ar at 773−1073 K using nickel dichloride as a catalyst precursor. X-ray diffraction (XRD), scanning electron microscopy (SEM), Raman spectrometry (Raman), a vibrating-sample magnetometer (VSM), and nitrogen adsorption/desorption were used to investigate the effects of the pyrolysis temperature and catalyst contents on the preparation of the aligned CNTs. As results, the as-obtained CNTs had an outer diameter of 30 nm, a wall thickness of 10 nm, and a length of about 50 μm, and their aspect ratio was high up to 1500. The aligned CNTs containing 0.75 wt% Ni prepared at 973 K exhibited good adsorption performance for methylene blue (MB); furthermore, benefiting from the special magnetic properties of residual Ni catalysts, the as-obtained CNTs could be easily magnetically recycled from the treated solution after adsorption.

## 1. Introduction

The recycling of waste commercial plastic and the development of an efficient method to recycle waste plastics is now a significant problem. Until now, numerous disposal methods for waste polyolefin have been developed, such as recycling, landfills, and incineration. Nevertheless, only a small amount of the plastic is actually recycled. Although some of the plastic can be handled by landfills, most plastics are non-degradable, and the method also occupied vast land resources. On the other hand, using incineration to dispose of waste plastics is not an efficient solution because it causes secondary pollution. Hence, it is still extremely important to develop a facile, economically feasible, and sustainable method to dispose of waste plastics and transform them into highly valuable products.

Carbon nanotubes (CNTs) have attracted significant attention in the fields of energy storage [1], electrochemical supercapacitors [2], field-emitting devices [3], solar cells [4], and composite materials [5]. Many methods, including an electric arc discharge method [6], a laser vaporization method [7], catalytic chemical vapor deposition (CVD) [8], and flame synthesis [9], have been developed to synthesize CNTs. Among the above-mentioned methods, CVD seems to be the most promising method for the mass production of CNTs at present. Unfortunately, conventional CVD methods suffer from various disadvantages, including employing high-purity light hydrocarbons as carbon sources [10] (such as methane, ethane, ethylene, etc.), resource-intensive production processes, and energy consumption [11].

Considering the high hydrogen and carbon content and high energy density, the production of CNTs from polyolefins and other organic waste provides a sustainable solution to both the disposal method of the waste and the mass production of carbon nanomaterials. For example, Lu et al. [12] synthesized entangled carbon nanotubes/fibers from fructose with an average diameter of 100 nm and a length of ~4 mm. Kong et al. [13] synthesized helical and straight CNTs with diameters ranging from 20 to 60 nm via catalytic pyrolysis of polyethylene (PE) in an autoclave at 973 K. Jiang et al. [14] synthesized multi-walled CNTs with diameters ranging from 50 to 60 nm through catalytic decombustion of polypropylene using nickel compounds (such as Ni_2_O_3_, NiO, Ni(OH)_2_, and NiCO_3_·2Ni(OH)_2_) as catalyst precursors in the presence of organic-modified montmorillonite at 900–1100 K. Zhang et al. [15] synthesized jumbled CNTs in the form of microspheres with diameters from 5.5 to 7.5 μm through catalytic pyrolysis of polypropylene and maleated polypropylene using Fe(C_5_H_5_)_2_ as a catalyst precursor at 973 K in an autoclave. Yen et al. [16] synthesized CNTs with diameters ranging from 25 to 90 nm and a length of 1 μm by using polyethylene as the carbon source and MgO and Fe(NO_3_)_3_ as catalysts at 1023–1123 K. Although these results were interesting, some significant issues remain to be solved: (1) Relatively expensive catalysts were used, and the manufacturing process was complicated; (2) the best growth parameters in terms of catalyst contents and pyrolysis temperature were still unclear; (3) the CNTs were curved and entangled together, and the quality of CNTs needed to be improved.

In the present paper, arrays of CNTs with a high aspect ratio (up to 1500) were obtained from low-cost waste polyethylene with nickel dichloride, which is much cheaper than nickel oxide, and ferrocene as catalysts; the adsorption performance for methylene blue (MB) of the as-obtained CNTs was investigated, and the effects of the Ni content and reaction temperature on the growth of the CNTs were also studied in detail.

## 2. Experimental

### 2.1. Materials and Preparation

Waste polyethylene powders (PE; Shanghai RunwenMaterial Co. Ltd., Shanghai, China.), nickel dichloride (NiCl_3_·6H_2_O; SinopharmChem. Co. Ltd., Shanghai, China), and nickel nitrate (Ni(NO_3_)_2_·6H_2_O; SinopharmChem. Co. Ltd., Shanghai, China) were used as raw materials. These chemicals were used directly without further purification.

The typical process of CNT synthesis can be described as follows: First, prescribed amounts of nickel dichloride (the weight ratios between Ni and waste polyethylene were 0.25, 0.50, 0.75, and 1.00 wt%) were dissolved into 10 mL of ethanol; the solution was then subjected to a magnetic stirrer at 298 K (room temperature). Then, the formed solution was carefully instilled into 10 g of powdery waste polyethylene to obtain the reactant precursors supporting with various amounts of nickel dichloride. The prepared precursors were initially desiccated at 323 K for 24 h in an oven, and 2 g of the resultant NiCl_3_/PE composite powder was placed in an alumina crucible and inserted into an alumina-tube furnace and then heated at a given temperature between 773 and 1073 K with a heating rate of 5 K/min before being held for 2 h in a flowing argon atmosphere (99.999 vol% pure).

### 2.2. Characterization

The carbon yield of the pyrolyzed waste polyethylene was calculated using the following equation:

(1) $$C=1-\frac{(m_{2}-m_{3})-m_{4}}{m_{1}-m_{4}}\times 100\%,$$

where *C* is the carbon residue ratio, *m*_1_ represents the weight of the NiCl_3_/PE composite powder, *m*_2_ is the weight of the NiCl_3_/PE composite powder and alumina crucible before pyrolysis, *m*_3_ is the weight of the NiCl_3_/PE composite powder and alumina crucible after pyrolysis, *m*_2_ − *m*_3_ is the weight loss of the waste polyethylene precursor before and after pyrolysis, and *m*_4_ is the weight of the Ni catalyst in the added nickel dichloride hexahydrate.

Crystalline phases of the as-obtained powders were analyzed by X-ray diffraction (XRD) using a Philips X′Pert Pro diffractometer (PANalytical, Hillsboro, The Netherlands) with the spectra ranging from 10° to 90° (2θ), scanning at a rate of 2°/min with Cukα radiation (λ = 0.1542 nm). Raman spectra were recorded once per sample using a Horiba Jobin-Yvon Labram-HR800 Raman spectrometer (Raman, Paris, France); a 532 nm diode laser was used with an integration time of 30 s, a spectral resolution of 1 nm, and an approximate power level of 2 mW. The morphologies of the samples were observed by means of a field-emission scanning electron microscope (FE-SEM; Nova400NanoSEM, 15 kV, Philips, Amsterdam, The Netherlands) and transmission electron microscope (TEM; JEM-2100UHRSTEM, 200 kV, JEOL, Tokyo, Japan) equipped with an energy dispersive spectrometer (EDS, Penta FET X-3 Si (Li)). N_2_ adsorption/desorption isotherms were recorded on an Autosorb-1 (USA) accelerated surface area and porosimetry instrument. The surface area was calculated from the adsorption branch of the isotherms using non-local density functional theory. The magnetic properties were examined by using a vibrating-sample magnetometer (VSM; Quantum Design, Inc., San Diego, CA, USA,) operating at room temperature (298 K).

### 2.3. Measurement of Adsorption Capacity

The adsorption isotherms of the as-obtained samples were examined by using MB as the adsorbate. For a typical adsorption measurement, a total of 20 mg of the as-obtained CNT powders was added into a beaker containing 50 mL of MB aqueous solution at concentrations ranging from 0 to 50 mg/L. The suspension was then stirred at a rate of 150 rpm at 288 K for 30 min. Then the concentration of the dye solution after centrifugation was examined using a ultraviolet–visible spectrophotometer (UV-Vis; Shimadzu UV-3600, Kyoto, Japan).

The adsorption kinetics of MB on the final samples was also investigated. Typically, 100 mg of the as-obtained CNTs was added to a MB dye aqueous solution (100 mL, 50 mg/L) in a beaker. The suspension was then subjected to magnetic stirring at a rate of 150 rpm at 288 K, and 5 mL of the suspension was taken from the beaker at 5 min time intervals and centrifuged to analyze the concentration of MB using a UV-Vis spectrophotometer; the adsorption intensity of the MB solutions at 664 nm was used for calculation.

## 3. Results and Discussion

### 3.1. Effects of Pyrolysis Temperature on the Preparation of Carbon Nanotubes from Waste Polyethylene

**Figure 1:**
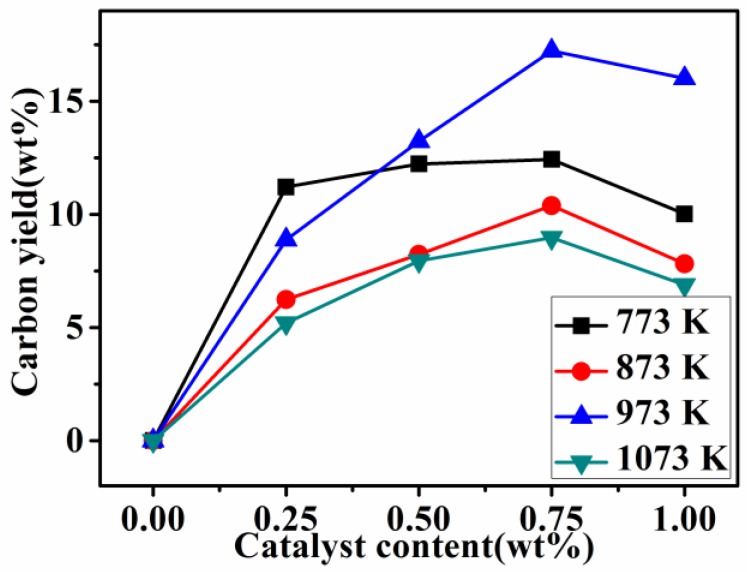
Carbon yield ratios of final product samples pyrolyzed at various temperatures with different catalyst contents.

Figure 1 shows the carbon yield ratios of the final product samples pyrolyzed at various temperatures with different catalyst contents. The carbon yield ratios of the final product samples adding different contents of the catalyst were much higher than the samples without the addition of the catalyst regardless of the heating treatment temperature. The highest carbon yield ratio of the sample was 17 wt% for the sample with 0.75 wt% Ni added, pyrolyzed at 973 K. This indicated that the addition of a Ni catalyst can greatly increase the conversion ratio of carbonaceous gases into solid carbon and that the present process is more environmentally friendly.

**Figure 2:**
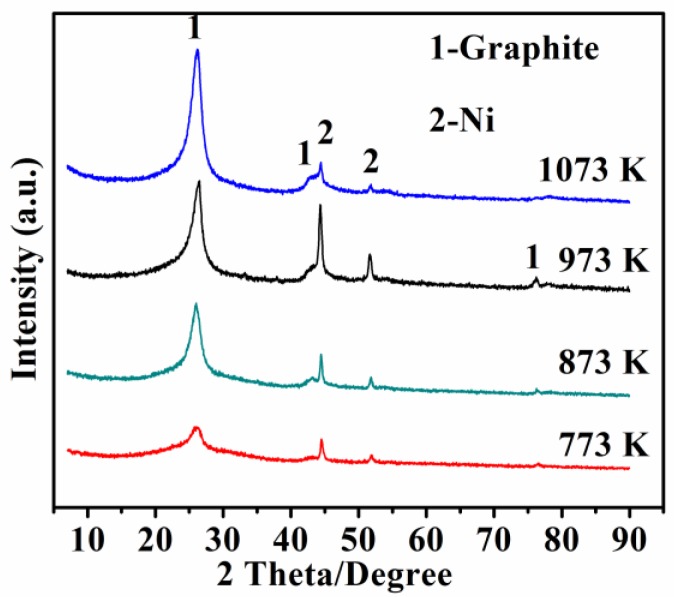
X-ray diffraction (XRD) patterns of samples resulting from 2 h pyrolysis of waste polyethylene with 0.75 wt% Ni catalyst at various temperatures.

XRD patterns of the samples prepared at 773 to 1073 K with 0.75 wt% Ni catalyst are presented in Figure 2. After 2 h of heat treatment at 773 K, visible broad diffraction peaks appeared at around 26°(2θ), which belonged to amorphous carbon in the sample. There were also two weak diffraction peaks appearing at 44.5° and 51.8°, which could be respectively assigned to the (111) and (200) planes of Ni (ICDD 01-089-7128). Additionally, the space group of Ni is Fm3m, and it is in fcc crystalline state. Previously published results demonstrated that the crystalline state of Ni has no effect on its magnetic properties [17].

Increasing the temperature up to 873 K, the relative intensity of graphite diffraction changed slightly. While increasing the temperature up to 973 K, the intensity of the carbon diffraction peaks increased again. Moreover, as the temperature increased to 1073 K, a sharp diffraction peak appeared at about 26°, indicating that the carbon was of a high graphitization degree, which matched well with the characteristic (002) plane of graphite (ICDD 01-075-1621). In addition, two other diffraction peaks were observed at 44.6° and 78.0°, respectively corresponding to the (101) and (110) planes of graphite.

**Figure 3:**
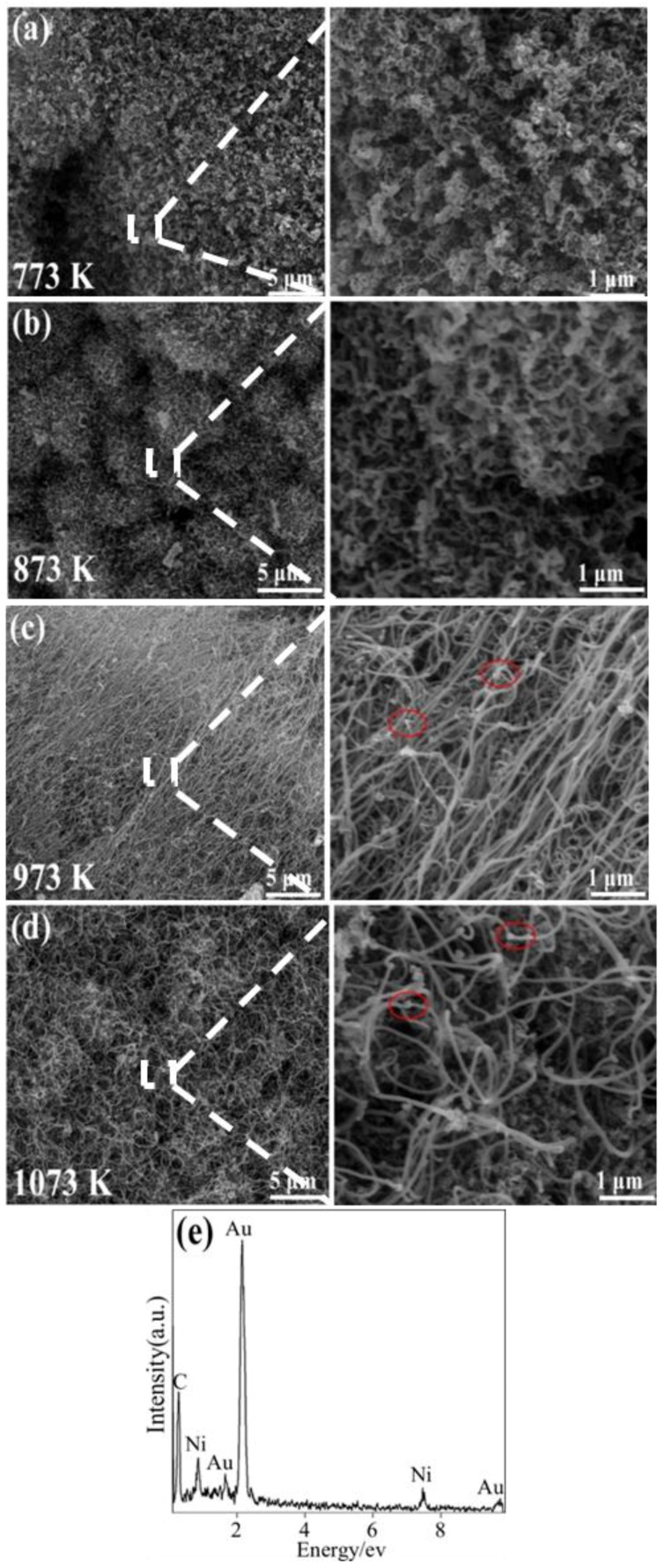
Scanning electron microscopy (SEM) images of the samples via pyrolysis of waste polyethylene with 0.75 wt% Ni catalyst at 773 K (**a**); 873 K (**b**); 973K (**c**); and 1073 K (**d**); and EDS of the nanoparticles in the area marked by the circles in Figure 3c in (**e**).

Figure 3 presents SEM images of the as-obtained samples resulting from the pyrolysis of waste polyethylene at 773, 873, 973, and 1073 K with the 0.75 wt% Ni catalyst, revealing that the morphologies of the final-product samples were clearly influenced by the reaction temperature. A few short CNTs were obtained at 773 K (Figure 3a). Increasing the temperature up to 873 K (Figure 3b), some curved and entangled CNTs were formed. However, when further increasing the temperature up to 973 K (Figure 3c), large amounts of straight CNTs were formed with diameters of 40 nm, lengths of 60 μm, and aspect ratios of 1500. Some nanoparticles with a size of 30 nm (shown in red circles in Figure 3c) were also seen at the tips and inners of partial CNTs. As confirmed by EDS results (in the area marked by the circles in Figure 3c), the nanoparticles with a size of 30 nm were ascribed to Ni (Figure 3e), implying that the tip-growth mechanism plays a key role during the formation of CNTs from pyrolyzed waste polyethylene. Moreover, as the temperature increased to 1073 K, the yield of CNTs decreased significantly, and some aggregated metal particles (80 nm) were observed in the sample (Figure 3d), indicating that a higher temperature (1073 K) was unfavorable to the formation of CNTs. According to the SEM observations above, it could be concluded that the optimal reaction temperature for the formation of CNTs in the present paper was about 973 K, which is consistent with the XRD results shown in Figure 2.

### 3.2. Effect of Ni Concentration on the Preparation of CNTs from Waste Polyethylene

**Figure 4:**
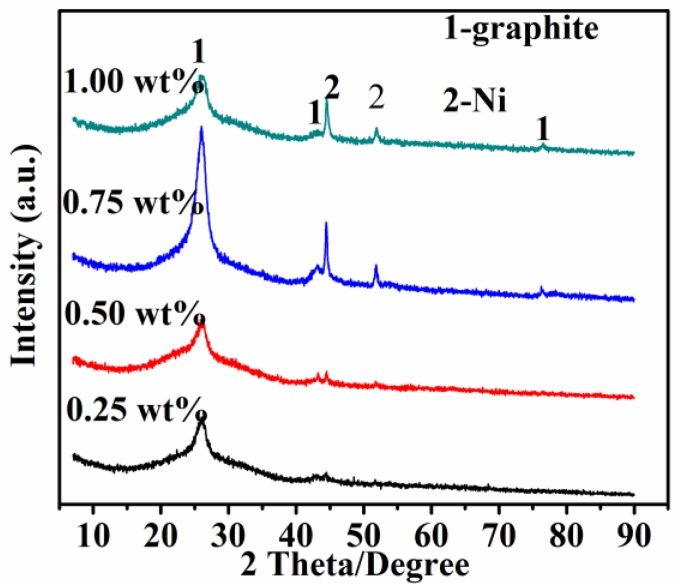
X-ray diffraction (XRD) patterns of product samples resulting from pyrolysis of waste polyethylene with various amounts of catalyst at 973 K.

Presented in Figure 4 are XRD patterns of the final product samples resulting from the pyrolysis of waste polyethylene with different contents of Ni added at 973 K. There was a broad diffraction peak of C and two weak diffraction peaks corresponding to Ni appearing in the sample containing 0.25 wt% Ni. Increasing the amount of the catalyst up to 0.50 wt%, the intensity of the diffraction peak of C did not change significantly, while the intensity of the diffraction peak of Ni increased slightly, indicating that such an amount of Ni was unfavorable for CNT growth. Increasing the amount of the catalyst to 0.75 wt% Ni, the intensity of the diffraction peak of C became the strongest, indicating the highest degree of carbon crystallization. At the same time, the intensity of the Ni diffraction peak also became sharp and strong, indicating the formation of more catalysts. Upon increasing the amount of Ni to 1.00 wt%, Ni diffraction peaks decreased again, which may be attributable to the aggregation and deactivation of the catalysts, revealing that a higher catalyst content was excessive for the preparation of CNTs. These above-mentioned experiments indicated that the catalyst content of 0.75 wt% Ni was appropriate for the formation of CNTs in the present paper.

**Figure 5:**
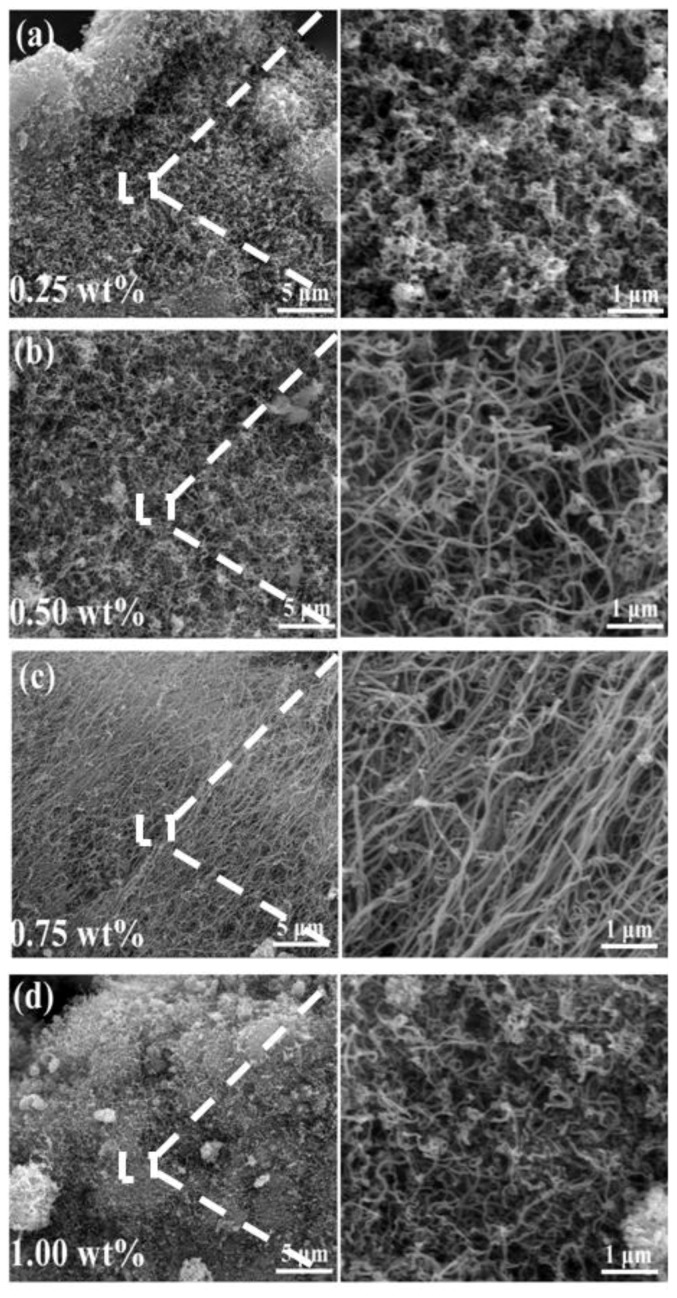
Scanning electron microscopy (SEM) images of final product samples resulting from pyrolysis of waste polyethylene at 973 K when adding different amounts of Ni catalysts: 0.25 wt% (**a**); 0.50 wt% (**b**); 0.75 wt% (**c**); and 1.00 wt% (**d**).

Figure 5 presents the SEM images of the product samples whose XRD patterns are shown in Figure 4, suggesting that the morphologies and yields of the CNTs were affected significantly by the amount of Ni catalyst. As shown in Figure 5a, a small amount of tangled and shorted CNTs were observed in the powders containing 0.25 wt% Ni catalyst, revealing that such an amount of Ni was not sufficient for the formation of high-quality CNTs. By increasing the Ni catalyst content to 0.50 wt%, the yield of the tubes increased observably, and massive tangled CNTs with a slight curve were formed in the sample (Figure 5b). Further increasing the amount of the catalyst up to 0.75 wt% Ni, more CNTs were formed in the sample (Figure 5c). In addition, the CNTs became straighter and the surface became smoother compared to those shown in Figure 5b. Upon further increasing the content of Ni to 1.00 wt%, the diameter of the as-formed CNTs increased, the yield decreased, and the array of CNTs was also destroyed (Figure 5d). Some larger catalyst particles were observed on the surface of the substrate. As is well known, the size of the catalyst is a major factor in determining its catalytic performance: a large (small) metal particle formed during the pyrolysis process can result in a relatively large (small) diameter of CNTs [18]. On the other hand, reasonable aggregation of Ni nanocatalysts increased with the increasing amount of Ni added, which decreased the catalytic activity of the catalysts [19,20]. Thus, high catalyst addition was also unnecessary for the effective formation of CNTs. It should be emphasized here that, as indicated in Figure 5c, straight and array-shaped CNTs with a high aspect ratio of up to 1500 were formed in the sample. In contrast, Wu et al. [21] also obtained CNTs with a length of 1 μm, 30 nm diameters, and 10 nm wall thicknesses from waste plastics, but the aspect ratios were 10 times lower than in this work. Zhuo et al. [22] prepared CNTs with diameters ranging from 30 to 85 nm and lengths of typically 1–5 μm via pyrolysis and combustion of polyethylene, but some curved and entangled CNTs were observed. On the basis of the above results, it could be assumed that 0.75 wt% Ni was optimal for the CNTs’ growth in the present paper.

**Figure 6:**
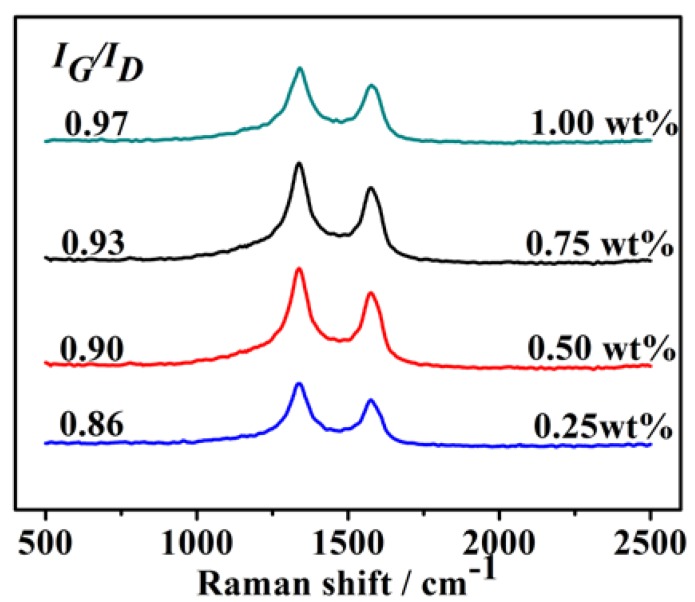
Raman spectra of product samples resulting from pyrolysis of waste polyethylene with various amounts of catalyst at 973 K.

Additionally, Raman spectra were obtained to further characterize the degree of graphitization of products with various amounts of Ni catalysts (Figure 6). In all the samples, the peak at about 1590 cm^−1^ (G-band) corresponded to the splitting of the *E*_2g_ stretching mode for hexagonal graphite, and the D-band (between 1305 and 1345 cm^−1^) was associated with the vibration of carbon atoms with dangling bonds in the plane terminations of disordered graphite or glassy carbons, indicating structural defects, vacancies, and carbonic impurities (amorphous carbon, glassy carbon, etc.) [23]. The intensity ratio of peak D to G (*I*_G_/*I*_D_) is commonly used to clarify the degree of graphitization of CNTs, and the *I*_G_/*I*_D_ ratio indicates information about the crystallinity of CNTs [24]. *I_G_/I_D_* ratios of the samples from waste polyethylene pyrolysis with various catalyst amounts calculated on the basis of the Raman spectra decreased in the following order: 1.00 wt% Ni (*I*_G_/*I*_D_ = 0.97) > 0.75 wt% Ni (*I*_G_/*I*_D_ = 0.93) > 0.50 wt% Ni (*I*_G_/*I*_D_ = 0.90) > 0.25 wt% Ni (*I*_G_/*I*_D_ = 0.86). This indicated that CNTs from waste polyethylene pyrolysis with 1.00 wt% Ni had a relatively faultless structure and a low density of lattice defects.

### 3.3. Transmission Electron Microscopy and High-Resolution Transmission Electron Microscopy Characterization

**Figure 7:**
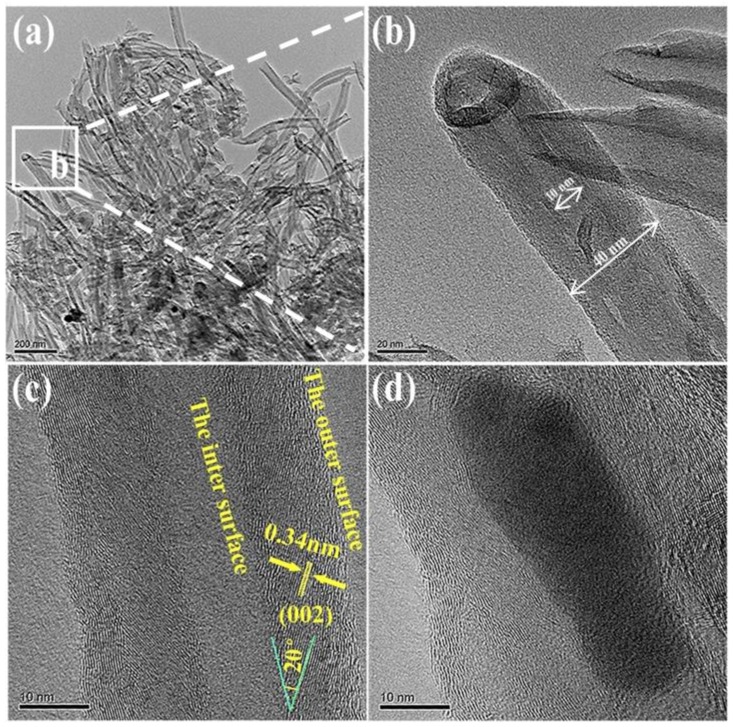
Transmission electron microscope (TEM) and high-resolution transmission electron microscope (HRTEM) images of product samples resulting from pyrolysis of waste polyethylene under optimal conditions: (**a**) low-magnification TEM; (**b**) an individual carbon nanotube (CNT) with a cap-shaped structure; (**c**) HRTEM image of an individual CNT, and (**d**) Ni catalyst encapsulated in a CNT.

Additionally, TEM and a high-resolution transmission electron microscope (HRTEM) analyses were also carried out to further characterize the morphologies and microstructures of the as-obtained CNTs synthesized under the optimal conditions (using 0.75 wt% Ni at 973 K) (Figure 7). CNTs with 30 nm in diameter and tens of micrometers in length were observed and are shown in Figure 7a. Additionally, as shown in the HRTEM images (in the area marked by the dotted square in Figure 7a), a “cap-shaped” ending at the tip of the CNTs was also observed, as shown in Figure 7b. TEM images (Figure 7b) implied that a Ni catalyst particle was located at the tip point of the CNTs, which provided forceful evidence for the tip-growth mechanism in the present CNT formation [25]. Most of the catalyst particles detached from the tip of the CNTs; thus, it was difficult to measure the Ni catalyst particle sizes. However, on the basis of the size of the holes resulting from the detaching of the Ni catalyst (Figure 7b), it could be estimated that the size of the catalyst was about 20 nm. A HRTEM image (Figure 7c) confirmed that the CNTs were well graphitized in the sample. The outer and inner diameters of the as-obtained CNTs were 30 and 10 nm, respectively. Moreover, the graphene layers that were oblique to the CNT axis at angles of 21°–25° (Figure 7c) were relatively straight, long, and smooth. On the basis of this, the as-obtained CNTs could be defined as cup-stacked carbon nanotubes (CS-CNTs) [26] that consisted of many truncated conical graphene layers. The interlayer spacing between the graphitic layers was measured as 0.34 nm, which matched well with the standard graphitic interlayer spacing of the (002) plane (0.34 nm); the numbers of walls were statistically mainly in the range of 30–50. In addition, a typical irregular bullet-shaped Ni catalyst on the inside of the hollow-centered CNTs is shown in Figure 7d; this was likely attributable to the coalescence and reconstruction of Ni catalyst nanoparticles during the pyrolysis of waste polyethylene [27].

It is generally accepted that the random chain scission of waste polyethylene products will lead to lower yields of aromatics and gas products and higher yields of alkenes with long chains [28,29]. It can be reasonably considered that aromatics and light hydrocarbons were the major carbon source for the growth of the Ni-catalyzed CNTs. As discussed above and in previously published literature [30,31], the formation mechanism of the present CNTs prepared via Ni-catalyzed pyrolysis carbonization of waste polyethylene is proposed and described as follows:(1) A NiCl_2_ catalyst precursor is gradually reduced to metallic Ni nanoparticles, contributing to the growth of CNTs; (2) carbon feedstock decomposes into carbon atoms on the surface of the Ni nanoparticles, and when the dissolving of carbon atoms in the catalysts reaches supersaturation, a graphitic monolayer ring is formed; (3) the graphitic ring grows in the axial direction while its diameter reduces, and the carbon-cap sprouts out; and (4) the CNT is extended in the axial direction when a new layer of carbon grows between the first layer and the particle.

**Figure 8:**
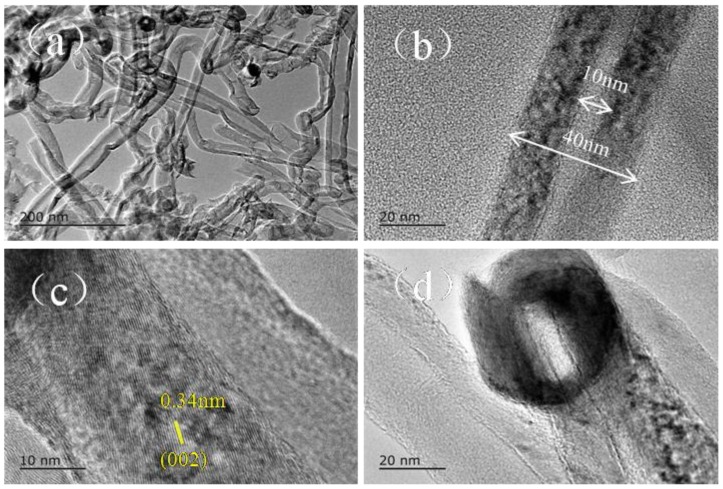
Transmission electron microscope (TEM) and high-resolution transmission electron microscope (HRTEM) images of samples prepared under 2h pyrolysis at 973 K using 0.75 wt% Ni(NO_3_)_2_ as a catalyst precursor: (**a**) low-magnification TEM; (**b**) TEM of an individual carbon nanotube (CNT); (**c**) HRTEM image of an individual CNT; and (**d**) the top of CNTs.

As discussed above, the NiCl_2_ catalyst precursor plays a key role in the synthetic reactions. Cl^−^ is known to induce structural defects in the graphitic lattice, as reported in many papers [26,32,33]. To elucidate the influence of the presence of Cl^−^ on the synthesis of CNTs, further samples were prepared under 2h of pyrolysis at 973 K using 0.75 wt% Ni(NO_3_)_2_ as a catalyst precursor. As shown in Figure 8, CNTs with 40 nm in diameter and tens of micrometers in length were observed and are shown in Figure 8a,b. CS-CNTs (Figure 8c) with a cap-shaped ending (Figure 8d) were also observed; the diameters of the synthesized CNTs were 30–60 nm, and the lengths were several microns. The interlayer spacing between the graphitic layers was measured as 0.34 nm, consistent with the standard graphitic interlayer spacing of the (002) plane (0.34 nm). These similar results to those of samples using the NiCl_2_ catalyst precursor indicate that the formation of CNTs was not significantly affected by the usage of the NiCl_2_ catalyst in the present paper. This was considered to be mainly relevant for the very low content of the added Cl^−^ ions (0.4 wt%).

**Figure 9:**
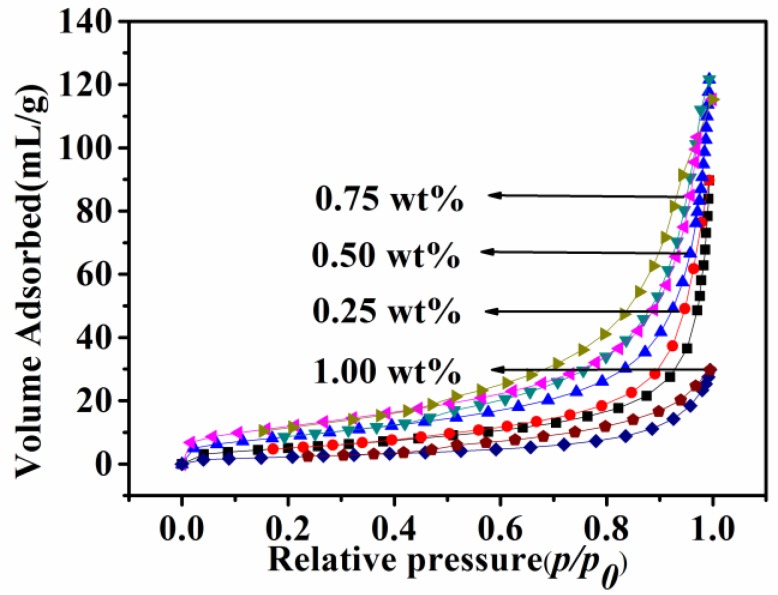
Nitrogen adsorption/desorption isotherms of carbon nanotubes (CNTs) prepared with different catalyst contents.

Nitrogen adsorption/desorption measurements were taken to determine the specific surface area of CNTs obtained with different Ni catalyst amounts from 0.25 to 1 wt% at 873 K. As presented in Figure 9, the resultant CNTs exhibited a typical type III isotherm without a hysteresis loop under a relative pressure (*P*/*P*_0_) of between 0.1 and 1.0, which indicated that the CNTs had a non-porous surface. Calculated from the adsorption branch of the isotherms using non-local density functional theory, the specific surface areas of the CNTs prepared with 0.25, 0.5, 0.75, and 1 wt% Ni catalysts were respectively calculated as 19.8, 33.1, 44.4, and 8.9 m^2^/g. 

**Figure 10:**
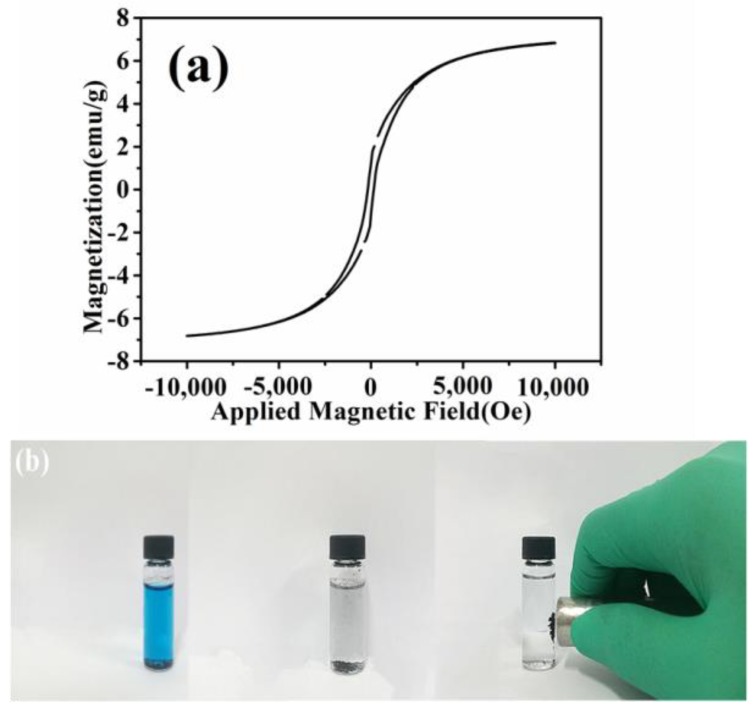
(**a**) The magnetic hysteresis loop of carbon nanotubes (CNTs) resulting from waste polyethylene; (**b**) photographs of the magnetic separation of CNTs from an aqueous dispersion.

The magnetic properties of the CNTs resulting from the pyrolysis of waste polyethylene were measured at room temperature (300 K) within the field of −10,000 to 10,000 Oe. The magnetic hysteresis loop shown in Figure 10a indicates the ferromagnetic behavior of the as-obtained CNTs, and the saturation magnetizations (*Ms*) of 6.84 emu∙g^−1^ could be ascribed to the existence of Ni nanoparticles in the CNTs. As a result, the CNTs distributed in water dispersion could be easily collected under an applied magnetic field, which should be advantageous for their recycling and reuse for adsorbing dye pollutants from water (Figure 10b).

### 3.4. Adsorption Capacity of Samples

**Figure 11:**
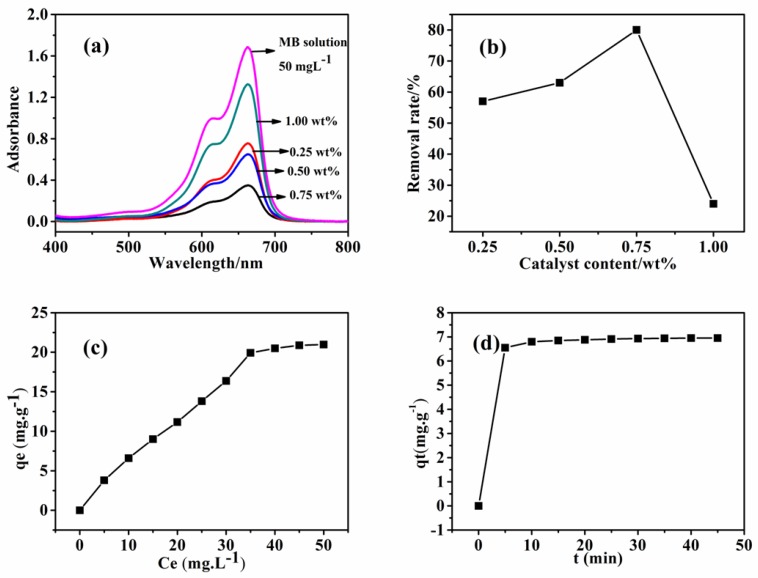
Adsorption capacity for methylene blue (MB) of the as-obtained carbon nanotubes (CNTs): (**a**) UV-Vis of CNTs obtained at different catalyst contents and (**b**) removal rate for MB; (**c**) adsorption isotherms and (**d**) adsorption kinetics of CNTs obtained with 0.75 wt% Ni catalyst.

The magnetic separation properties and the specific surface areas make the as-obtained CNTs an ideal absorbent to remove organic dye pollutants from water [34,35], and the relationship between the absorbent concentrations and removal efficiency of the CNTs is discussed. The adsorption capacities of the as-obtained CNTs for MB were identified by UV-Vis and are shown in Figure 11. It was observed that the as-obtained CNT adsorption capacities were different from those prepared under various catalyst contents (Figure 11a); moreover, the adsorption capacity order of the samples prepared under different reaction conditions decreased as follows: 0.75 wt% Ni > 0.5 wt% Ni> 0.25 wt% Ni> 1 wt% Ni. The dependence of the specific surface area of the samples obtained with different catalyst contents on the adsorption capacity (Figure 11a,b) indicated that the samples prepared with the 1 wt% Ni catalyst had the lowest adsorption capacity; in contrast, the samples obtained with the 0.75 wt% Ni catalyst had the highest adsorption capacity, consistent with the Nitrogen adsorption/desorption measurements result shown in Figure 9. Therefore, this confirmed that the CNTs’ adsorption capacity mainly depended on their specific surface area. The adsorption capacity increased as the specific surface area increased [36]. The adsorption capacity was also assigned to the π–π stacking between MB and the π-conjugation regions on the CNT surfaces [37]. The adsorption capacity for MB of the CNTs obtained with the 0.75 wt% Ni catalyst was found and is shown in Figure 11c,d; the adsorption isotherms of the samples were simulated separately using the Langmuir and Freundlich models:

Langmuir isotherm:

(2) $$q_{e}=\frac{Q_{0}bc_{e}}{1+c_{e}^{1/n}}$$

Freundlich isotherm:

(3) $$q_{e}=k_{F}c_{e}^{1/n}$$

Here, *q*_e_ (mg/g) is the equilibrium adsorption amount, *Q*_0_ (mg/g) is the maximum adsorption amount, *b* (L/mg) is the constant term related to the energy of adsorption, *c*_e_ (mg/L) is the equilibrium concentration of the organic dye solution, and k_F_ and n are the Freundlich constants. The fitted parameters and *R*^2^ values as given in Table 1 on the basis of the Langmuir and Freundlich models were respectively 0.961 and 0.984, suggesting that the Freundlich isotherm was more accurate. The maximum monolayer adsorption capacity (*Q*_0_) of the as-obtained CNTs was calculated as 33.1 mg/g.

To further understand the adsorption mechanism, the following pseudo-first-order and pseudo-second-order kinetic models were used to investigate the kinetics of MB removal on the as-obtained CNTs:

Pseudo-first-order model:

(4) $$q_{t}{=q}_{e}{(1-e}^{{-k}_{1}t})$$

Pseudo-second-order model:

(5) $$\frac{t}{q_{t}}=\frac{1}{k_{2}q_{e}{}^{2}}+\frac{t}{q_{e}}$$

Here, *q*_t_ (mg/g) is the adsorption amount at time *t*, and *k*_1_ and *k*_2_ (g·mg^−1^·min^−1^) are respectively the pseudo-first-order and pseudo-second-order rate constants. The calculated kinetic parameters and correlation coefficients (*R*^2^) are also listed in Table 1, indicating that the pseudo-second-order model showed good linearity, with a *R*^2^ value of above 0.999. Therefore, it could be concluded that the pseudo-second-order model fit better with the experimental results.

## 4. Conclusions

We present a facile method to fabricate CNT arrays with a high aspect ratio of up to 1500 from waste polyethylene through a one-step catalytic pyrolysis method by using relatively cheap nickel dichloride as a catalyst precursor; the as-obtained CNTs were about 30–50 nm in diameter and tens of microns in length. XRD, SEM, Raman, and TEM results revealed that the catalyst precursor nickel dichloride was reduced to metal Ni, which afterwards acted as a catalyst for the growth of CNTs from waste polyethylene pyrolysis. The optimal parameters for the CNTs were 2 h of pyrolysis at 973 K using a 0.75 wt% Ni catalyst. Additionally, the as-obtained CNTs showed good adsorption capacity for MB; moreover, the obtained CNTs could be easily recycled from the treated dye pollutant dispersion by a magnet owing to the special magnetic properties of residual Ni nanoparticle catalysts.

## Figures and Tables

**Table 1:** Kinetics parameters calculated on the basis of Equations (2) and (3) for methylene blue (MB) adsorption of carbon nanotubes (CNTs) prepared with 0.75 wt% Ni catalysts.

Langmuir			Freundich			Pseudo-First-Order			Pseudo-Second-Order		
*Q*_0_	*b*	*R* ^2^	k_F_	n	*R* ^2^	*q*_e_	*k*_1_	*R* ^2^	*q*_e_	*k*_2_	*R* ^2^
33.10	0.068	0.961	1.056	1.26	0.984	15.6	0.41	0.987	16.5	0.1433	0.999
